# Simple Detection of Pigment Red 53 as a Hazardous Substance in Cosmetic Preparation Using a Polymer Combination of Polystyrene (PS) and Polymethylmethacrylate (PMMA)

**DOI:** 10.3390/molecules27249016

**Published:** 2022-12-17

**Authors:** Rimadani Pratiwi, Syifa Amanda, Aliya Nur Hasanah

**Affiliations:** Department of Pharmaceutical Analysis and Medicinal Chemistry, Faculty of Pharmacy, Universitas Padjadjaran, Bandung 45363, Indonesia

**Keywords:** pigment red 53, polystyrene, PMMA, indicator strip

## Abstract

Pigment red 53 is a synthetic dye that has been banned in cosmetic products due to the possibility of causing blood disorders and spleen sarcoma. The indicator strip employs qualitative analysis methods that are simpler, easier, and quicker than an instrumental analysis. The indicator strip is made of a polymethylmethacrylate (PMMA) and polystyrene (PS) mixture using a reagent blending method with specific reagents of concentrated sulfuric acid (H_2_SO_4_), concentrated hydrochloric acid (HCl), or 10% sodium hydroxide (NaOH). Pigment red 53 detections with an indicator strip are based on the occurrence of a specific color change reaction between the reagent and pigment red 53 through sulfonation with concentrated H_2_SO_4_, neutralization with 10% NaOH, and reaction of pigment red 53’s azo group with concentrated HCl. PMMA was made with a concentration of 5% (w/t), and mixtures of PS:PMMA 1:2, 1:3, and 1:4 had solvent-to-specific reagent ratios of 60:40, 80:20, and 90:10. The best results were obtained for PMMA-H2SO4 (90:10), PMMA-HCl (80:20), and PMMA-NaOH (60:40), with the lowest detection limits equaling 20 ppm, 50 ppm, and 20 ppm, respectively. Meanwhile, the best PS:PMMA (1:4)-based indicator strips obtained were PS:PMMA-H_2_SO_4_ (90:10), PS:PMMA-HCl (80:20), and PS:PMMA-NaOH (60:40), with the lowest detection limits being 20 ppm, 10 ppm, and 20 ppm, respectively. All indicator strips are stable for at least 80 days. Indicator strips can be used as a simple and applicable method for detecting pigment red 53 in cosmetic products with a good performance.

## 1. Introduction

Cosmetics are materials or preparations that are applied to the outside of the human body to clean, scent, change appearance, and/or improve body odor, as well as protect/maintain the body [1]. According to the Association of Indonesian Cosmetics Companies and Associations, cosmetics sales reached USD 7.45 million in 2021, a 7% increase from the previous year [2]. Due to potential market opportunities, dyes in the cosmetic industry are frequently abused by substituting synthetic textile dyes for legal cosmetic dyes [3,4]. On November 14, 2018, four of the six findings contained pigment red 53 in lipstick products, according to Public Warning No. B-HM.01.01.1.44.11.18.5410 concerning cosmetics containing hazardous materials [5]. The number of illegal cosmetics discovered and/or containing prohibited/hazardous materials has increased from 0.65% to 0.74% in the last five years, with a total value of IDR 185.9 billion [6,7].

Pigment red 53 (C.I. 15585, D&C Red No. 8) is a red β-naphthol pigment lake, which is a fast solvent and has high staining strength [8]. In general, pigment red 53 is used as an ink in large-scale printing processes [9]. It is also recommended for powder coatings, PCV, rubber, PP, PS, and PE [10]. The long-term use of pigment red 53 can result in toxicity, which manifests as blood disorders and spleen sarcoma [9]. Pigment red 53 has been banned in the United States since 1988, the European Union since 1988 [9], and Indonesia since 1990 via the Director-General of Drug and Food Control under decree number 00386/C/SK/II/90 concerning certain dyes declared as hazardous substances in medicine, food, and cosmetics. Included in this were also pigment orange 5 (C.I. 12075, D&C Orange No. 17), pigment red 53:1 (C.I. 15585:1, D&C Red No. 9), rhodamine B (C. I. 45170, D&C Red No. 9), and solvent red 49 (C.I. 45170:1).

To date, the methods developed for determining pigment red 53 have been extremely limited, and necessitate the use of instruments such as TLC, UV-vis spectrophotometry, HPLC, NMR, and LC/MS-MS [9]. However, these methods are less effective and efficient when applied in the field. The community requires an alternative method to determine the presence of pigment red 53 without bringing it to a laboratory. As a result, the indicator strip is a qualitative analysis method that is simple and easy to use for onsite analysis [11]. The indicator strip was developed by using polymethylmethacrylate (PMMA) and a mixture of polystyrene (PS) and PMMA containing a specific reagent, which reacts with the analyte and produces a specific color associated with that analyte. The indicator strip would be designed using the reagent blending method, and the resulting indicator strips can be characterized to include accuracy, sensitivity, stability, and selectivity tests.

## 2. Results and Discussions

### 2.1. Sample Collection and Preparation

All samples have an organoleptic red color, and three out of nine samples are legal products according to the registration number checked on the Food and Drug Administration of Indonesia’s website. DMF was used as a solvent extractor due to it being more cost-efficient [12] than the mixed solvent N,N-dimethylformamide—orthophosphoric acid (95:5) [13]. Furthermore, DMF is frequently used as a solvent in a variety of synthetic procedures, including the production of dyes. This is supported by a broad liquid temperature range, good chemical and thermal stability (even at its boiling point, 153⁰C), high polarity, and a broad solubility range for organic and inorganic compounds [14,15].

### 2.2. Selection of Specific Chemical Reagent for Pigment Red 53

The reagents used in this study were concentrated sulfuric acid (reagent A), concentrated hydrochloric acid (reagent B), 10% sodium hydroxide solution (reagent C), and 10% ammonia solution (reagent D). The pigment red 53 turned purplish-red by adding reagent A, very light purple by adding reagent B, pale pink by adding reagent C, and brownish-orange by adding reagent D [16]. As shown in Table 1, reagents A, B, and C produce the same color according to the literature. Chemical reagents were used based on the structure of pigment red 53. Based on the structure, pigment red 53 has both a phenol and azo group. Reagent A was chosen because it undergoes reversible sulfonation with the phenol present in pigment red 53 to form phenol sulfonic acid, producing a purplish-red color. Through neutralization, the phenol group in pigment red 53 also reacts with reagent C to produce pale pink sodium phenoxide [17,18,19]. Pigment red 53’s azo group reacts with re-agent B to produce light purple [20], whereas barium ions react with reagent D to produce a brownish-orange color [21]. The reagents used here resulted in the same color obtained in the reference results. As seen in Table 1, reagent D did not produce a brownish-orange color, which was most likely due to the need for solution preparation with H_2_S gas [22]. Furthermore, the reaction of ammonia with barium hydroxide was very slow [23]. Hence, reagent D did not meet the criteria to be chosen as a reagent to be incorporated into the strip. Based on these findings, reagents A, B, and C continued to be used in the next step.

### 2.3. Development of Indicator Strip from PMMA and A Mixture of PS and PMMA Using a Reagent Blending Method

Reagent blending is accomplished by dissolving PMMA polymer and a mixture of PS and PMMA in a suitable solvent, and then mixing it with specific chemical reagents. PS and PMMA are chloroform- and ethyl acetate-soluble [24]. The solvent ethyl acetate was chosen based on the Hildebrand solubility parameter (δ). The closer the Hildebrand solubility parameter between the solvent and solute, the better the solvent is at dissolving the solute [25]. Ethyl acetate as a solvent has a closer Hildebrand solubility parameter (δ) to PS and PMMA as solutes. The Hildebrand solubility parameter (δ) of ethyl acetate as a solvent was 9.1 (cal1/2 cm−3/2), while the Hildebrand solubility parameters of PS and PMMA as solutes were 9.13 (cal1/2 cm−3/2) and 9.3 (cal1/2 cm−3/2), respectively [26].

The indicator strip’s pore density is proportional to the polymer concentration. The 2.5% concentration produced a brittle indicator strip, while the 7.5% concentration resulted in indicator strips with overly tight pores [27]. As a result, the 5% concentration was chosen to produce a strong indicator strip with less dense pores. PMMA indicator strips were prepared at a concentration of 5% by shaking for 10 min with a magnetic stirrer at 5 rpm. The optimum PS/PMMA ratio is 1:4 with agitation using a magnetic stirrer at 5 rpm for 48 min. When PS:PMMA ratios of 1:2 and 1:3 are compared, indicator strips are less homogeneous, brittle, and thin, making them difficult to use at operating pressures exceeding 1 bar.

The solvent–chemical reagent ratio is 90:10 for reagent A, 80:20 for reagent B, and 60:40 for reagent C. Variations in the composition of the solvent-specific chemical reagent ratio are affected by the strength of the acid because PMMA is more acid-resistant than PS. According to the results of the indicator strip test, the indicator strip shows the appropriate color change with the 1000 ppm pigment red 53 standard solution, as shown in Figure S1 in the Supplementary Materials; Table 2 shows the detailed results of the indicator strip.

### 2.4. Performance Test of the Indicator Strip

#### 2.4.1. Sensitivity and Stability Test

A sensitivity test was performed to determine the indicator strip’s sensitivity in detecting the minimum limit of pigment red 53 concentration. PMMA-H_2_SO_4_ (90:10), PMMA-HCl (80:20), and PMMA-NaOH (60:40) can detect pigment red 53 with the lowest detection limits of 20 ppm, 50 ppm, and 20 ppm, respectively. Meanwhile, PS:PMMA-H_2_SO_4_ (90:10), PS:PMMA-HCl (80:20), and PS:PMMA-NaOH (60:40) can detect pigment red 53 with the lowest detection limits of 20 ppm, 10 ppm, and 20 ppm, respectively.

A stability test was performed to determine the indicator strip’s stability and resistance to detecting pigment red 53 at time intervals after reagent blending. The stability test of the indicator strip was performed every day until the indicator strip did not change color, or produce a positive result. The results show that all indicator strips were stable for up to 80 days.

#### 2.4.2. Accuracy Test

**Figure 1:**
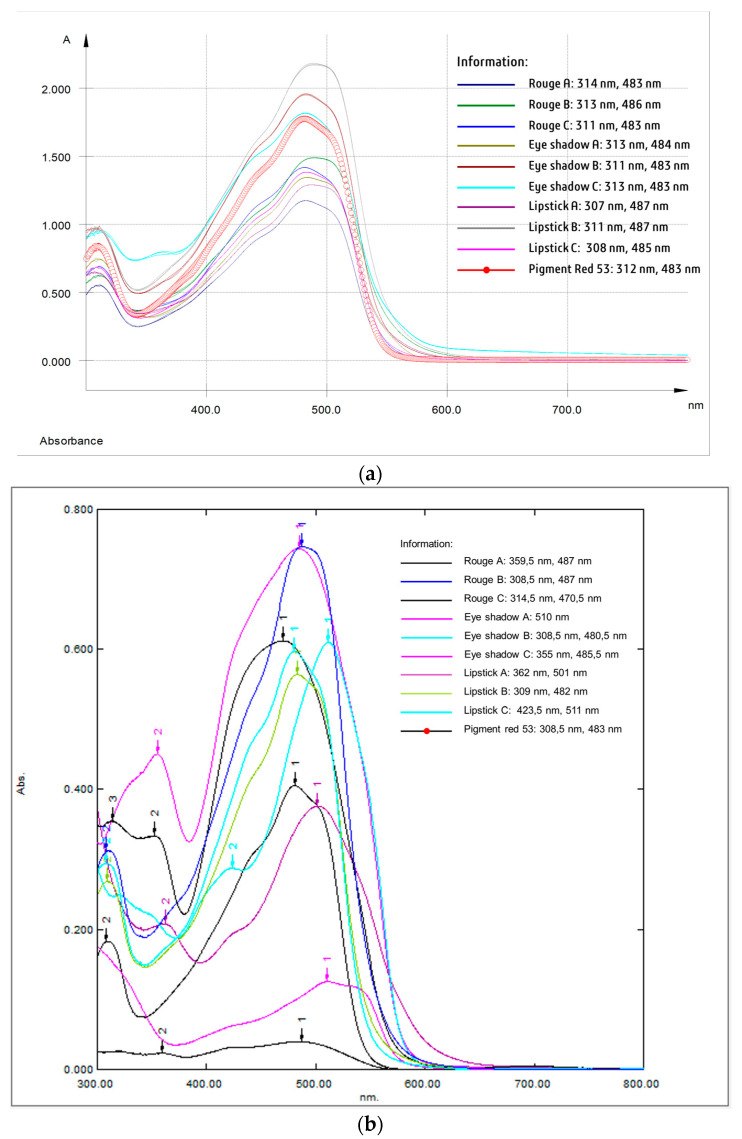
(**a**) UV-visible spectrum profile of spiked samples, (**b**) UV-visible spectrum profile of pure samples.

The accuracy test was performed to compare the presence of pigment red 53 in cosmetic samples detected by UV-vis spectrophotometry with indicator strips. As shown in Figure 1a, when the sample was spiked with pigment red 53, the maximum wavelength was similar to the pigment red 53 standard, with a wavelength of 312 nm and 483 nm and a shifted wavelength of 3 nm, which still meets the requirements [28]. This indicates that the extraction processes were successful. The presence of pigment red 53 in the pure sample was confirmed using a UV-vis spectrophotometry analysis. As shown in Figure 1b, of the nine samples, only three showed positive results. The samples included eyeshadow B, lipstick B, and rouge B, with concentrations of 14.82 ppm, 18.64 ppm, and 13.98 ppm, respectively.

The positive samples from the UV analysis were then analyzed with an indicator strip to confirm the indicator strip’s performance. Based on the data, the concentration of pigment red 53 in the samples was below the lowest detection limit of the indicator strip, except for PS:PMMA (1:4)-HCl. This was most likely due to the presence of a matrix effect. However, the indicator strip remains accurate due to its capability of producing a suitable color change when reacting with spiked samples, and being selective to rhodamine B, as shown in Table 3.

#### 2.4.3. Selectivity Test

The selectivity test was performed by using rhodamine B, a dye that is often also used for cosmetics. Rhodamine B was prepared in a DMF solvent and as shown in Table 3. When rhodamine B reacted with the indicator strip it produced a negative result. According to its structure, rhodamine B lacks a phenol or an azo group that will react with specific chemical reagents [29].

### 2.5. Characterization of Indicator Strip

#### 2.5.1. Scanning Electron Microscope-Energy Dispersive X-ray Spectroscopy (SEM-EDX)

**Figure 2:**
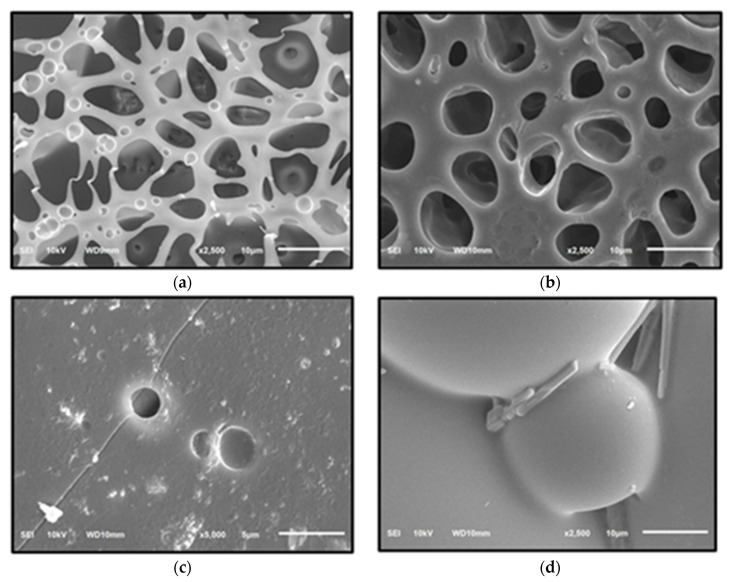
PMMA microstructures: (**a**) PMMA; (**b**) PMMA-H_2_SO_4_; (**c**) PMMA-HCl; (**d**) PMMA-10% NaOH.

**Figure 3:**
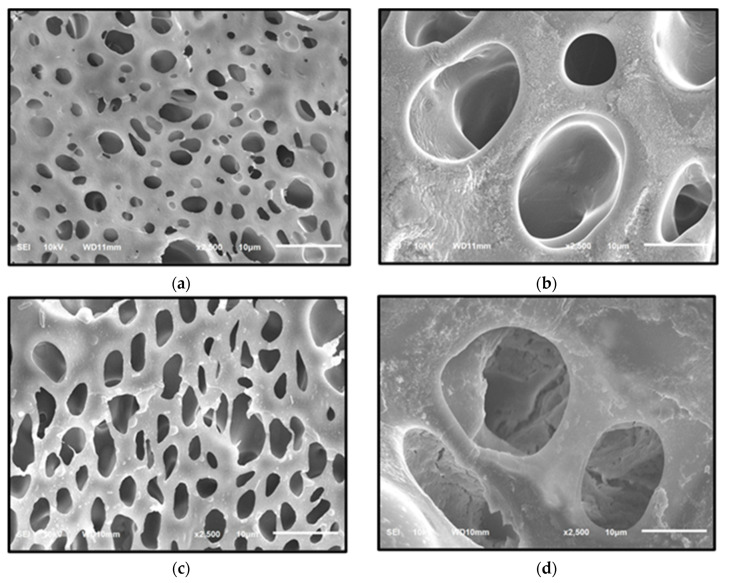
PMMA microstructures: (**a**) PS:PMMA; (**b**) PS:PMMA-H_2_SO_4_; (**c**) PS:PMMA-HCl; (**d**) PS:PMMA-10% NaOH.

The morphology of the indicator strip was examined using SEM at magnifications of 2500× or 5000×, as shown in Figure 2 and Figure 3. According to the SEM analysis, PMMA has a more homogeneous structure, which is characterized by the formation of relatively regular intersegment chains and cavities [30]. Meanwhile, a 1:4 PS:PMMA mixture forms an inhomogeneous structure, despite PS being more acid-resistant than PMMA [11]. This can be attributed to an increase in the viscosity of the solution caused by the presence of PS in the mixture, resulting in shear stress. Shear stress causes PS to split from the PMMA matrix, resulting in various cavities [31] and surface cracks [11].

The elemental composition of specific chemical reagents in the indicator strip is measured using Energy Dispersive X-ray Spectroscopy (EDX), as shown in Table 4, while EDX spectra are shown in Figures S2 to S9 in the Supplementary Materials. The reaction between the elements in the specific chemical reagent and the pigment red 53 functional group causes the color change in the indicator strip. Based on the EDX analysis, specific chemical reagents were mixed into the indicator strip, which is distinguished by the presence of constituent elements [24].

#### 2.5.2. Infrared (IR) Spectroscopy

**Figure 4:**
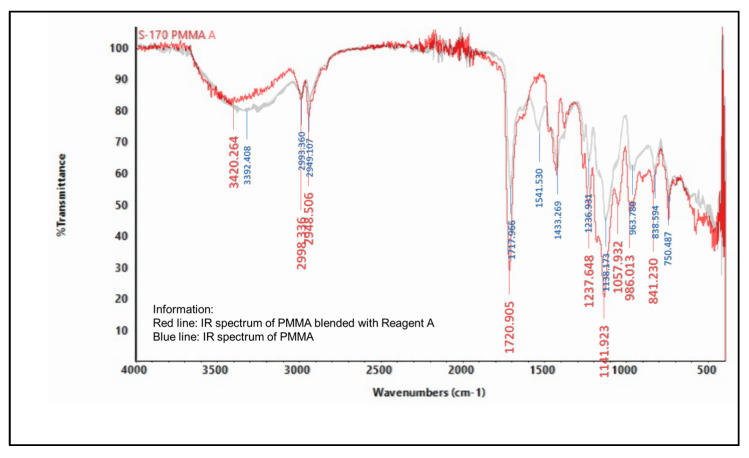
IR spectrum comparison of pure PMMA and blended PMMA with reagent A (concentrated H_2_SO_4_).

**Figure 5:**
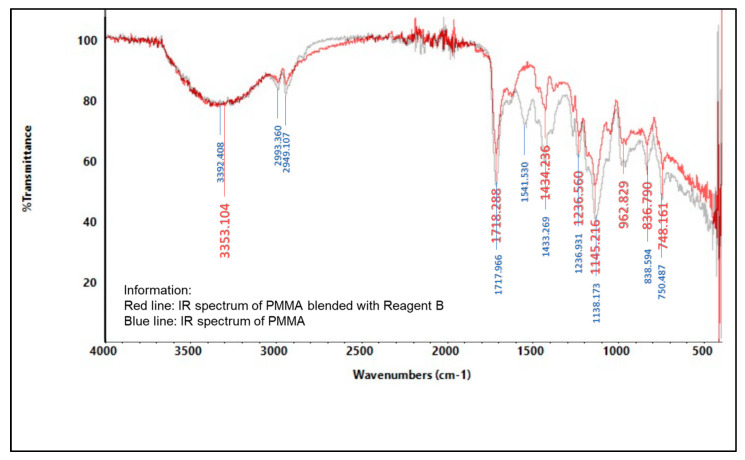
IR spectrum comparison of pure PMMA and blended PMMA with reagent B (concentrated HCl).

**Figure 6:**
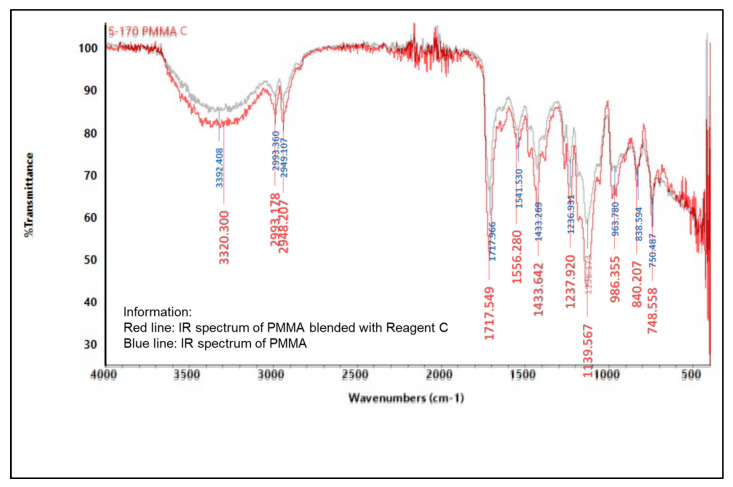
IR spectrum comparison of pure PMMA and blended PMMA with reagent C (10% NaOH).

**Figure 7:**
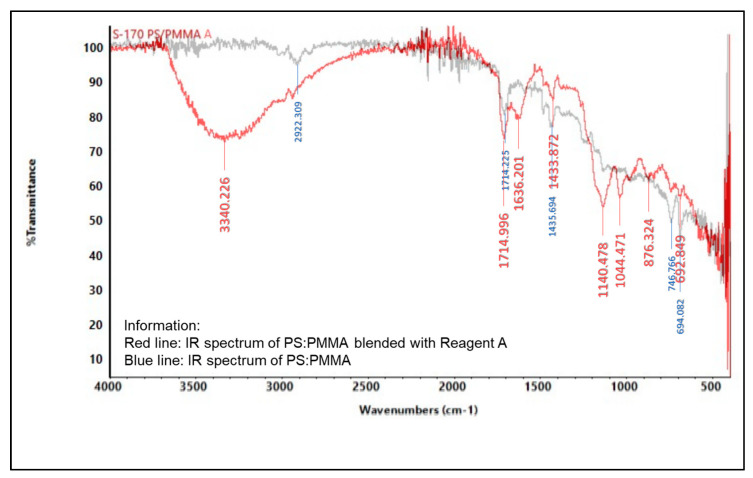
IR spectrum comparison of pure PS:PMMA and blended PMMA with reagent A (concentrated H_2_SO_4_).

**Figure 8:**
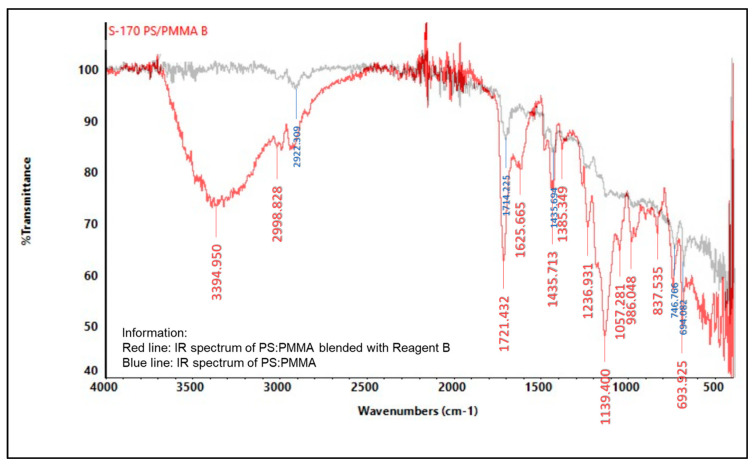
IR spectrum comparison of pure PS:PMMA and blended PMMA with reagent B (concentrated HCl).

**Figure 9:**
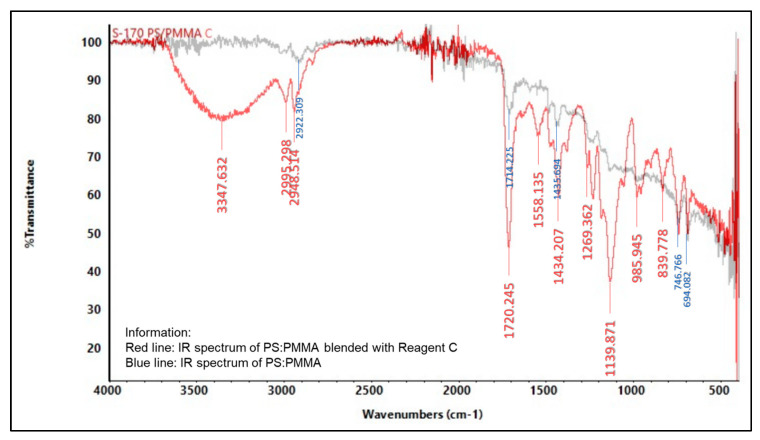
IR spectrum comparison of pure PS:PMMA and blended PMMA with reagent C (10% NaOH).

An infrared spectrophotometer was used to analyze the presence of various functional groups in the polymer, as shown in Figure 4, Figure 5, Figure 6, Figure 7, Figure 8 and Figure 9. Detailed information is shown in Tables S1 to S6 in the Supplementary Materials. PS ring deformation with a medium and sharp peak at 694 cm^−1^ was observed in the primary PS functional group, while C=O stretching vibrations with a weak and sharp peak at 1717 cm^−1^ were observed in the primary PMMA functional group [32].

According to the findings, several changes in peak intensity and peak shift are due to specific reagents, which blended into the indicator strip [32]. When the PMMA-based indicator strip is blended with reagent A (concentrated sulfuric acid), peak intensity will increase at 1700–900 cm^−1^ range. When the PMMA and PS:PMMA mixture-based indicator strip is blended with reagent B (concentrated hydrochloric acid), peak intensity will change at 1700 cm^−1^. Reagent C (sodium hydroxide) will increase peak intensity to 1700–1100 cm^−1^ for the PMMA-based indicator strip, and 1700 cm^−1^ for the PS:PMMA mixture-based indicator strip. Furthermore, the acid and base content of reagents A, B, and C on PS:PMMA mixture-based indicator strips can eliminate peaks at 2900 cm^−1^ due to chemical interactions.

## 3. Materials and Methods

### 3.1. Chemicals and Materials

All chemicals used were of analytical grade and used without further purification. Ammonium hydroxide 25%, ethanol, ethyl acetate, hydrochloric acid, N,N-dimethylformamide (DMF), N-butanol, natrium hydroxide, sulfuric acid, and rhodamine B were purchased from Merck (Darmstadt, Germany). Pigment red 53 and Polymethylmethacrylate (PMMA) were obtained from TCI. Polystyrene (PS) was purchased from Sigma Aldrich (St. Louis, MO, USA).

### 3.2. Sample Collection and Preparation

The sample of cosmetic products was obtained from the Bandung area. The sample was selected based on the probability that it contained pigment red 53 in products such as eyeshadow, lipstick, and rouge [27,33]. There were nine products in the total sample, with three brands of each cosmetic. A 50 mg sample was then dissolved in 5 mL of DMF and sonicated for 30 min by heating and then filtered [13,15]. The sample was used as a pure test solution; meanwhile, the spiked test solution was made by spiking 1 mL of the pure test solution with 100 ppm pigment red 53, and adding up to 5 mL of DMF.

### 3.3. Selection of Specific Chemical Reagent for Pigment Red 53

The reagents used in this study were concentrated sulfuric acid, concentrated hydrochloric acid, 10% sodium hydroxide solution, and 10% ammonia solution, which resulted in color changes when reacting with pigment red 53 solutions. Pigment red 53 turned purplish-red with the addition of concentrated sulfuric acid, very light purple with the hydrochloric acid, pale pink with the 10% sodium hydroxide solution, and brownish-orange with the 10% ammonia solution [16]. Only positive color changed chemical reagents were chosen for the development of indicator strips with the reagent blending method.

### 3.4. Fabrication of Indicator Strip from PMMA and a Mixture of PS and PMMA Using the Reagent Blending Method

PMMA polymer and PS:PMMA polymer mixtures (1:2, 1:3, and 1:4) were prepared at concentrations of 5% in an ethyl acetate solvent with each chosen specific chemical reagent at a ratio of 60:40, 80:20, and 90:10. Each polymer solution was shaken with a magnetic stirrer at ±5 rpm until all the polymer was dissolved. Then, the polymer solution was poured onto the glass, which was coated with black duct tape as a barrier, and allowed to dry to make a membrane out of the polymer [27,33]. 

### 3.5. Performance Test of the Indicator Strip

At each optimum condition, the performance of the indicator strip was tested, including testing for sensitivity, accuracy, stability, and selectivity.

#### 3.5.1. Sensitivity Test

A 1 × 1 cm indicator strip was tested with various concentrations of pigment red 53 (5, 10, 15, 20, 30, 40, 50, 100, 250, 500, and 1000 ppm). The indicator strip’s lowest detection limit to detect pigment red 53 was observed when the indicator strip still produced a color change at the lowest concentration. 

#### 3.5.2. Accuracy Test

A 1 × 1 cm indicator strip was tested on samples known to contain pigment red 53 organoleptically, and was compared with UV-vis spectrophotometry (Analytik Jena Specord 200, Jena, Germany and Shimadzu UV-1800, Kyoto, Japan). For the UV-vis spectrophotometry, 1 mL of the pure sample (not spike) and spike sample solution were taken, diluted to 3 mL, and analyzed in the absorbance range of 300–800 nm [9]. The suitability of the instrument’s results and the indicator strip’s results were then assessed [33]. 

#### 3.5.3. Stability Test

A 1 × 1 cm indicator strip was tested at time intervals after blending. Stability was then observed until the indicator strip did not produce a color change or a positive result [27,33].

#### 3.5.4. Selectivity Test

A 1 × 1 cm indicator strip was tested on different red dyes, such as rhodamine B, to determine whether the specific chemical reagents contained in the indicator strip reacted with another compound besides pigment red 53 [33].

### 3.6. Characterization of Indicator Strip

The characterization of indicator strips was performed to determine whether the specific chemical reagents used have mixed well with the polymers [33].

#### 3.6.1. Scanning Electron Microscope-Energy Dispersive X-ray Spectroscopy (SEM-EDX)

Scanning Electron Microscope-Energy Dispersive X-ray Spectroscopy (SEM-EDX, JEOL JSM 6510 LA, Tokyo, Japan) was used in the characterization of the indicator strips to analyze their microstructure, and the integration of specific reagents on polymers [33] at 2500× and 5000× magnification.

#### 3.6.2. Spectrophotometer Infrared (IR) 

An infrared spectrophotometer (ATR Nicolet Summit BDM1910155, Thermo Fisher, Waltham, MA, USA) was used to analyze the functional groups of specific chemical reagents used in the indicator strips. A change in the intensity of the functional groups [33], as well as the addition of elements in the indicator strip after the mixing process, indicate the mixing of specific chemical reagents into the indicator strip.

## 4. Conclusions

An indicator strip based on polymethylmethacrylate-specific reagents and a mixture of polystyrene:polymethylmethacrylate-specific reagents can be used to detect pigment red 53 in cosmetics with a good performance. This method can be a simple and easy way to detect pigment red 53 in cosmetics for onsite analysis.

## Figures and Tables

**Table 1:** Specific chemical reagent color change with 1000 ppm pigment red 53 standard solution.

Reagent	Reagent Strength	Documentation	Result
A	Concentrated H_2_SO_4_	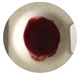	+
B	Concentrated HCl	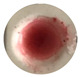	+
C	10% NaOH	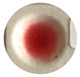	+
D	10% Ammonia	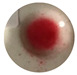	−

**Table 2:** Result of developing indicator strips from the PMMA and PS:PMMA mixture using a reagent blending method.

Polymer	Solvent and Reagent Ratio	Solvent	Reagent	Result	Time Reaction to Color Change (Second)
PMMA 5%	60:40	Ethyl acetate	A	**−**	−
	80:20			**−**	−
	90:10			+	25.41
	60:40		B	−	−
	80:20			+	10.33
	60:40		C	+	22.48
PS:PMMA (1:2)	90:10	Ethyl acetate	A	+	32.13
	80:20		B	+	22.69
	60:40		C	+	18.36
PS:PMMA (1:3)	90:10	Ethyl acetate	A	+	31.42
	80:20		B	+	17.41
	60:40		C	+	16.51
PS:PMMA (1:4)	90:10	Ethyl acetate	A	+	30.78
	80:20		B	+	10.14
	60:40		C	+	17.26

Information: (−): no polymer membrane was formed; (+): polymer membrane was formed; A: concentrated sulfuric acid; B: concentrated hydrochloric acid; C: 10% sodium hydroxide.

**Table 3:** Results of the selectivity test of indicator strips from the PMMA and PS:PMMA mixtures using the reagent blending method.

Indicator Strip Material	Reagent	Dye	
		Pigment Red 53	Rhodamine B
PMMA 5%	A	+	−
	B	+	−
	C	+	−
PS:PMMA (1:4)	A	+	−
	B	+	−
	C	+	−

Information: (−): no polymer membrane was formed; (+): polymer membrane was formed; A: concentrated sulfuric acid; B: concentrated hydrochloric acid; C: 10% sodium hydroxide.

**Table 4:** Elemental mass percentage in the indicator strip.

Indicator Strip	Element	%Mass
PMMA 5%	Carbon	67.24
	Oxygen	32.76
PMMA-H_2_SO_4_ 90:10	Carbon	41.73
	Oxygen	39.31
	Sulfur	18.96
PMMA-HCl 80:20	Carbon	61.99
	Oxygen	36.04
	Chlorine	1.97
PMMA-10% NaOH 60:40	Carbon	62.02
	Oxygen	36.46
	Sodium	1.52
PS:PMMA (1:4) 5%	Carbon	71.20
	Oxygen	28.80
PS:PMMA-H_2_SO_4_ 90:10	Carbon	40.59
	Oxygen	42.45
	Sulfur	16.96
PS:PMMA-HCl 80:20	Carbon	67.61
	Oxygen	28.62
	Chlorine	3.77
PS:PMMA-10% NaOH 60:40	Carbon	66.45
	Oxygen	29.79
	Sodium	3.76

## Data Availability

Data are available within the article and Supplementary Materials.

## Supplementary Materials

The following supporting information can be downloaded at: https://www.mdpi.com/article/10.3390/molecules27249016/s1, Figure S1. Color change on the optimum condition of indicator strip when react with pigment red 53; Figure S2. EDX Spectrum of PMMA 5%; Figure S3. EDX Spectrum of PMMA-H2SO4 (90:10); Figure S4. EDX Spectrum of PMMA-HCl (80:20); Figure S5. EDX Spectrum of PMMA-10% NaOH (60:40); Figure S6. EDX Spectrum of PS:PMMA (1:4) 5%; Figure S7. EDX Spectrum of PS:PMMA-H2SO4 (90:10); Figure S8. EDX Spectrum of PS:PMMA-HCl (80:20); Figure S9. EDX Spectrum of PS:PMMA-10% NaOH (60:40); Table S1: PMMA functional groups before and after blending with reagent A (concentrated sulfuric acid) comparison; Table S2: PMMA functional groups before and after blending with reagent B (concentrated hydrochloric acid) comparison; Table S3: PMMA functional groups before and after blending with reagent C (10% sodium hydroxide) comparison; Table S4: PS:PMMA functional groups before and after blending with reagent A (concentrated sulfuric acid) comparison; Table S5: PS:PMMA functional groups before and after blending with reagent B (concentrated hydrochloric acid) comparison; Table S6: PS:PMMA functional groups before and after blending with reagent C (10% sodium hydroxide) comparison.
