# Supplementary material for: Simple Detection of Pigment Red 53 as a Hazardous Substance in Cosmetic Preparation Using a Polymer Combination of Polystyrene (PS) and Polymethylmethacrylate (PMMA)
Source: Molecules. 2022 Dec 17;27(24):9016. doi: 10.3390/molecules27249016 (PMC9784189; doi:10.3390/molecules27249016)
Supplement: Supplementary file 1 [file molecules-27-09016-s001.zip › molecules-2045515-supplementary.pdf]

molecules

(Supplementary Material)

Simple Detection of Pigment Red 53 As a Hazardous Substance in Cosmetic Preparation using a Polymer Combination of Polystyrene (PS) and Polymethylmethacrylate (PMMA)

Rimadani Pratiwi *, Syifa Amanda and Aliya Nur Hasanah

Department of Pharmaceutical Analysis and Medicinal Chemistry, Faculty of Pharmacy, Universitas Padjadjaran, Bandung 45363, Indonesia
* Correspondence: rimadani.pratiwi@unpad.ac.id

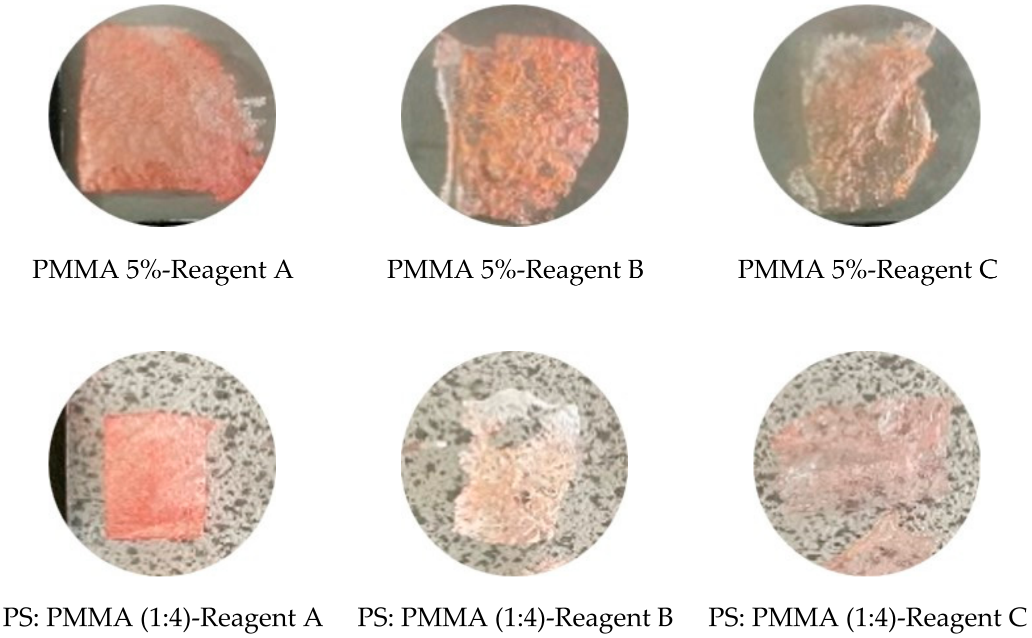

Figure S1. Color change on the optimum condition of indicator strip when react with pigment red 53

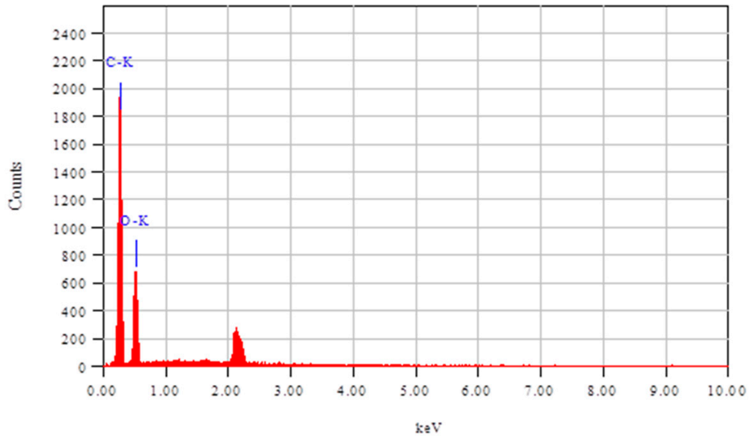

Figure S2. EDX Spectrum of PMMA 5%

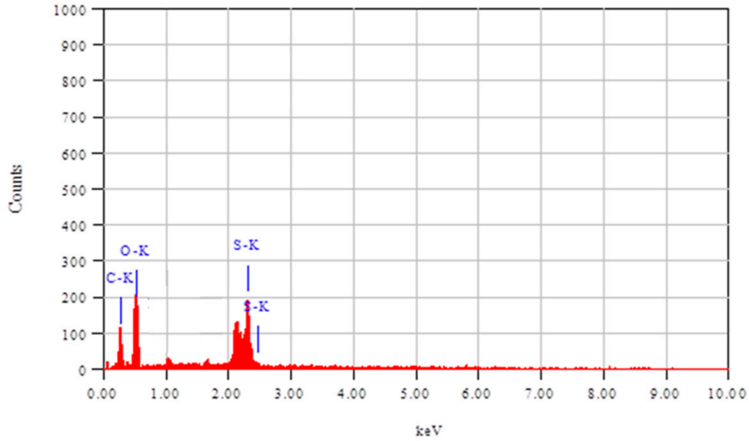

Figure S3. EDX Spectrum of PMMA-H2SO4 (90:10)

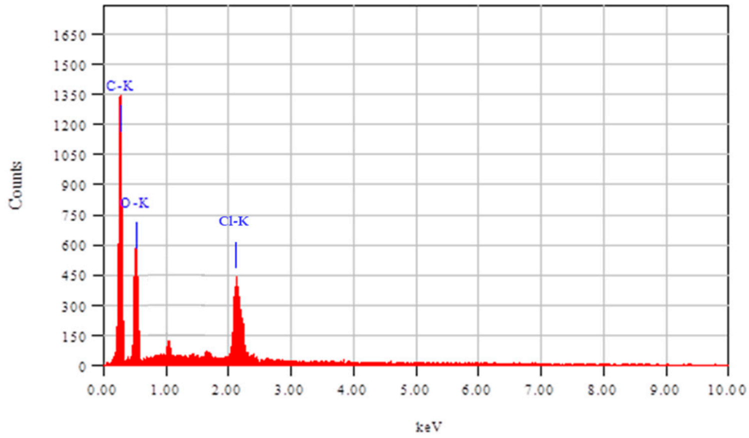

Figure S4. EDX Spectrum of PMMA-HCl (80:20)

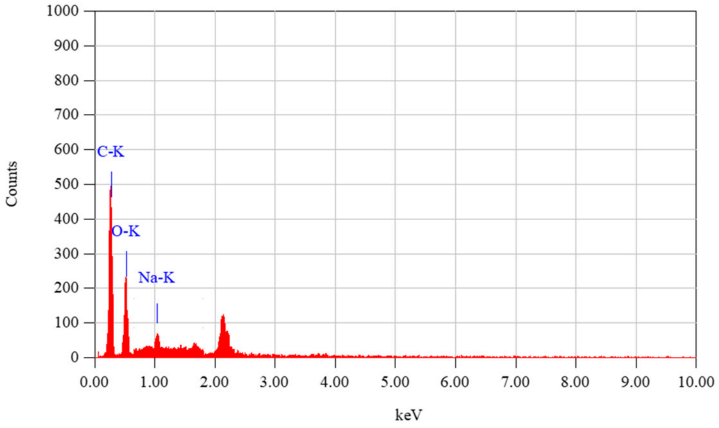

Figure S5. EDX Spectrum of PMMA-10% NaOH (60:40)

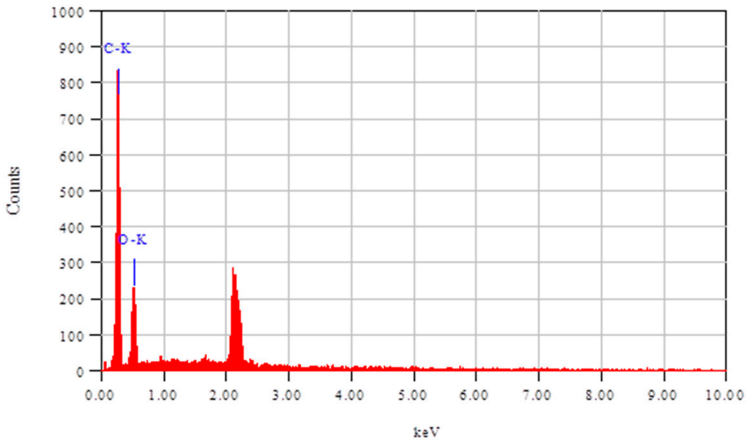

Figure S6. EDX Spectrum of PS:PMMA (1:4) 5%

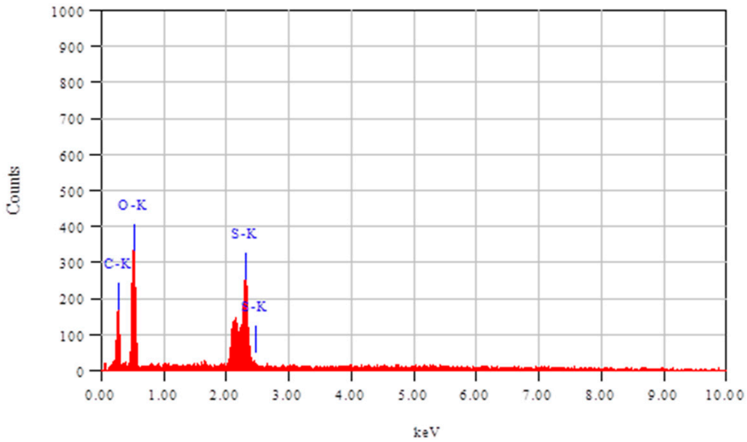

Figure S7. EDX Spectrum of PS:PMMA-H2SO4 (90:10)

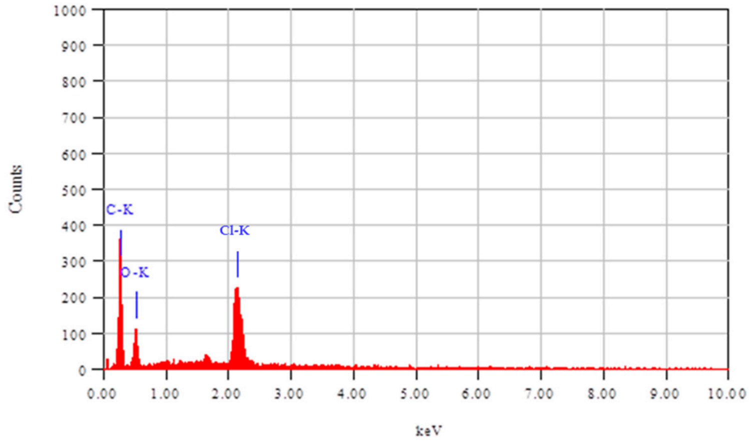

Figure S8. EDX Spectrum of PS:PMMA-HCl (80:20)

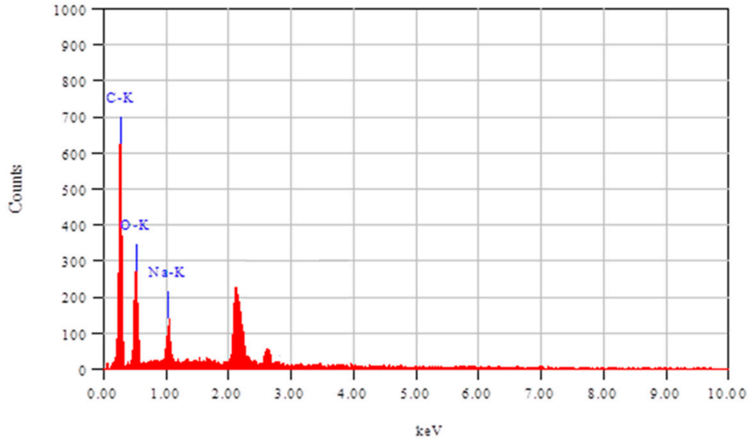

Figure S9. EDX Spectrum of PS:PMMA-10% NaOH (60:40)

IR Characterization Results of Indicator Strips Made from Pure PMMA and PMMA-Reagent Mixture

Table S1. PMMA Functional Groups Before and After Blending with Reagent A (Concentrated Sulfuric Acid) Comparison

PMMA			PMMA with Reagent A		
**Wavenumber (cm^{-1})**	**Intensity and Shape**	**Functional Group Analysis**	**Wavenumber (cm^{-1})**	**Intensity and Shape**	**Functional Group Analysis**
3392,408	Weak, broad	O-H stretching vibration	3420,264	Weak, sharp	O-H stretching vibration
2993,306	Weak, sharp	Asymmetric C-H stretching vibration	2998,336	Weak, sharp	Asymmetric C-H stretching vibration
2949,107	Weak, sharp	Symmetric C-H stretching vibration	3948,506	Weak, sharp	Symmetric C-H stretching vibration
1717,966	Weak, sharp	C=O stretching vibration from PMMA	1720,905	Strong, sharp	C=O stretching vibration from PMMA
1541,530	Weak, sharp	CH_3 Deformation	-	-	-
1433,569	Weak, sharp	C-CH_3 bending vibration	1434,562	Weak, sharp	C-CH_3 bending vibration
1236,931	Weak, sharp	Symmetric C-C-O stretching vibration	1237,648	Medium, sharp	Symmetric C-C-O stretching vibration
1138,173	Medium, sharp	Asymmetric C-O-C stretching vibration	1141,923	Strong, sharp	Asymmetric C-O-C stretching vibration
963,780	Weak, sharp	O-CH_3 rock vibration	968,013	Medium, sharp	O-CH_3 rock vibration
838,594	Weak, sharp	CH_2 rock vibration	841,230	Weak, sharp	CH_2 rock vibration
750,487	Medium, sharp	C-C stretching vibration	748,821	Medium, sharp	C-C stretching vibration

Information: (-) = No peak

Table S2. PMMA Functional Groups Before and After Blending with Reagent B (Concentrated Hydrochloric Acid) Comparison

PMMA			PMMA with Reagent B		
**Wavenumber (cm^{-1})**	**Intensity and Shape**	**Functional Group Analysis**	**Wavenumber (cm^{-1})**	**Intensity and Shape**	**Functional Group Analysis**
3392,408	Weak, broad	O-H stretching vibration	3343,104	Weak, broad	O-H stretching vibration
2993,306	Weak, sharp	Asymmetric C-H stretching vibration	-	-	-
2949,107	Weak, sharp	Symmetric C-H stretching vibration	2948,524	Weak, sharp	Symmetric C-H stretching vibration
1717,966	Weak, sharp	C=O stretching vibration from PMMA	1716,288	Weak, sharp	C=O stretching vibration from PMMA
1541,530	Weak, sharp	CH_3 Deformation	-	-	-
1433,569	Weak, sharp	C-CH_3 bending vibration	1434,236	Weak, sharp	C-CH_3 bending vibration
1236,931	Weak, sharp	Symmetric C-C-O stretching vibration	1236,560	Weak, sharp	Symmetric C-C-O stretching vibration
1138,173	Medium, sharp	Asymmetric C-O-C stretching vibration	1145,216	Medium, sharp	Asymmetric C-O-C stretching vibration
963,780	Weak, sharp	O-CH_3 rock vibration	962,829	Weak, sharp	O-CH_3 rock vibration
838,594	Weak, sharp	CH_2 rock vibration	836,790	Weak, sharp	CH_2 rock vibration

750,487	Medium, sharp	C-C stretching vibration	748,161	Weak, sharp	C-C stretching vibration

Information: (-) = No peak

Table S3. PMMA Functional Groups Before and After Blending with Reagent C (10% Sodium Hydroxide) Comparison

**PMMA**			**PMMA with Reagent C**		
**Wavenumber (cm^{-1})**	**Intensity and Shape**	**Functional Group Analysis**	**Wavenumber (cm^{-1})**	**Intensity and Shape**	**Functional Group Analysis**
3392,408	Weak, broad	O-H stretching vibration	3320,300	Weak, broad	O-H stretching vibration
2993,306	Weak, sharp	Asymmetric C-H stretching vibration	2993,178	Weak, sharp	Asymmetric C-H stretching vibration
2949,107	Weak, sharp	Symmetric C-H stretching vibration	2948,207	Weak, sharp	Symmetric C-H stretching vibration
1717,966	Weak, sharp	C=O stretching vibration from PMMA	1717,549	Strong, sharp	C=O stretching vibration from PMMA
1541,530	Weak, sharp	CH_3 Deformation	1556,280	Weak, sharp	CH_3 Deformation
1433,569	Weak, sharp	C-CH_3 bending vibration	1422,642	Medium, sharp	C-CH_3 bending vibration
1236,931	Weak, sharp	Symmetric C-C-O stretching vibration	1237,920	Weak, sharp	Symmetric C-C-O stretching vibration
1138,173	Medium, sharp	Asymmetric C-O-C stretching vibration	1139,567	Strong, sharp	Asymmetric C-O-C stretching vibration
963,780	Weak, sharp	O-CH_3 rock vibration	986,355	Medium, sharp	O-CH_3 rock vibration

838,594	Weak, sharp	CH_2 rock vibration	840,207	Weak, sharp	CH_2 rock vibration
750,487	Medium, sharp	C-C stretching vibration	748,558	Medium, sharp	C-C stretching vibration

IR Characterization Results of Indicator Strips Made from Pure PS:PMMA and PS:PMMA-Reagent Mixture

Table S4. PS:PMMA Functional Groups Before and After Blending with Reagent A (Concentrated Sulfuric Acid) Comparison

PS:PMMA			PS:PMMA with Reagent A		
**Wavenumber (cm^{-1})**	**Intensity and Shape**	**Functional Group Analysis**	**Wavenumber (cm^{-1})**	**Intensity and Shape**	**Functional Group Analysis**
2922,309	Weak, sharp	Methylene	-	-	-
1714,225	Weak, sharp	C=O stretching vibration	1724,996	Weak, sharp	C=O stretching vibration
1435,694	Weak, sharp	C-CH_3 bending vibration	1433,872	Weak, sharp	C-CH_3 bending vibration
746,766	Medium, sharp	C-H bending vibration	-	-	-
694,082	Medium, sharp	PS ring deformation	692,849	Medium, sharp	PS ring deformation

Information: (-) = No peak

Table S5. PS:PMMA Functional Groups Before and After Blending with Reagent B (Concentrated Hydrochloric Acid) Comparison

PS:PMMA			PS:PMMA with Reagent B		
**Wavenumber (cm^{-1})**	**Intensity and Shape**	**Functional Group Analysis**	**Wavenumber (cm^{-1})**	**Intensity and Shape**	**Functional Group Analysis**
2922,309	Weak, sharp	Methylene	-	-	-
1714,225	Weak, sharp	C=O stretching vibration	1721,432	Medium, sharp	C=O stretching vibration
1435,694	Weak, sharp	C-CH_3 bending vibration	1435,713	Weak, sharp	C-CH_3 bending vibration

746,766	Medium, sharp	C-H bending vibration	747,303	Medium, sharp	C-H bending vibration
694,082	Medium, sharp	PS ring deformation	693,925	Medium, sharp	PS ring deformation

Information: (-) = No peak

Table S6. PS:PMMA Functional Groups Before and After Blending with Reagent C (10% Sodium Hydroxide) Comparison

**PS:PMMA**			**PS:PMMA with Reagent C**		
**Wavenumber (cm^{-1})**	**Intensity and Shape**	**Functional Group Analysis**	**Wavenumber (cm^{-1})**	**Intensity and Shape**	**Functional Group Analysis**
2922,309	Weak, sharp	Methylene	-	-	-
1714,225	Weak, sharp	C=O stretching vibration	1721,432	Medium, sharp	C=O stretching vibration
1435,694	Weak, sharp	C-CH_3 bending vibration	1435,713	Weak, sharp	C-CH_3 bending vibration
746,766	Medium, sharp	C-H bending vibration	748,720	Medium, sharp	C-H bending vibration
694,082	Medium, sharp	PS ring deformation	696,066	Medium, sharp	PS ring deformation

Information: (-) = No peak
